# Seroprevalence of Hepatitis B Virus and Human Immunodeficiency Virus and associated factors among pregnant women attending antenatal care in selected general hospitals of Mekelle and Eastern Zone of the Tigray Region, northern Ethiopia

**DOI:** 10.1016/j.ijregi.2026.100909

**Published:** 2026-05-02

**Authors:** Senait Kebede Abadi, Kiflom Hagos, Mebrahtu Teweldemedhin Shfare, Angesom Kebede Abadi, Tadele Araya

**Affiliations:** 1 Department of Medical Microbiology and Immunology, Faculty of Medical Laboratory Sciences, College of Health Sciences, Mekelle University, Mekelle, Tigray, Ethiopia; 2 Department of Obstetrics and Gynecology, School of Medicine, College of Health Sciences, Mekelle University, Mekelle, Ethiopia

**Keywords:** Seroprevalence, HBV, HIV, Mekelle, Tigray, Ethiopia, Pregnant women

## Abstract

**Objectives:**

Hepatitis B virus (HBV) and human immunodeficiency virus (HIV) share common transmission risk factors and remain major causes of maternal morbidity and mortality, especially in low- and middle-income countries. However, updated local data on the burden of HBV and HIV in North Ethiopia are limited. This study aimed to assess the seroprevalence of HBV and HIV and associated factors among pregnant women attending antenatal care in selected general hospitals of Mekelle and the eastern zone of Tigray, northern Ethiopia.

**Methods:**

A hospital-based cross-sectional study was conducted from December 2024 to April 2025 in selected general hospitals of Mekelle and the eastern zones of Tigray. Sociodemographic risk factor data and blood samples were collected from 238 pregnant women. Rapid diagnostic tests were used to detect HBV and HIV, with confirmation by enzyme-linked immunosorbent assay.

**Results:**

The overall seroprevalence of HBV and HIV were 9.7% and 4.2%, respectively. Variables like older age (adjusted odds ratio [AOR] = 3.10, 95% confidence interval [CI]: 1.01-9.46, *P* = 0.047), history of liver disease (AOR = 23.67, 95% CI: 3.55-158.01, *P* = 0.001), history of abortion (AOR = 0.24, 95% CI: 0.06-0.98, *P* = 0.047), nose piercing (AOR = 3.80, 95% CI: 1.33-10.88, *P* = 0.013), and history of sexually transmitted infections (AOR = 6.03 95% CI: 1.56-23.35; *P* = 0.009) had significant associations with seroprevalence of HBV, while knowledge about transmission of HIV (exact odds ratio = 0.10, 95% CI: 0.02-0.72, *P* = 0.0219) was the only variable significantly associated with seroprevalence of HIV in this study.

**Conclusion:**

The overall seroprevalence of HBV and HIV among pregnant women attending antenatal care was high. Therefore, it is essential to implement effective prevention strategies to reduce the risk of mother-to-child transmission of these viral infections.

## Highlights

• Hepatitis B virus prevalence: 9.7%; human immunodeficiency virus prevalence: 4.2%; no co-infections detected.
• Hepatitis B virus risk increased with age >25 years (adjusted odds ratio [AOR] = 3.10), liver disease (AOR = 23.67), nose piercing (AOR = 3.80), and history of sexually transmitted infections (AOR = 6.03).
• Knowledge about human immunodeficiency virus transmission reduced infection odds by 90% (exact odds ratio = 0.10).
• Findings support targeted prevention mother-to-child transmission strategies.

## Introduction

Hepatitis B virus (HBV) attacks the liver and can cause both acute and chronic disease. The most common transmission mechanisms of the infection are from women to child during birth, in early childhood, through contact with blood or other body fluids during sex with a partner with the infection, and through unsafe injections or exposure to sharp instruments [1]. Chronic HBV infection may progress to liver cirrhosis and hepatocellular carcinoma, contributing substantially to preventable morbidity and mortality worldwide, despite the availability of safe and effective vaccines [1].

Human immunodeficiency virus (HIV) targets immune cells essential for host defense and continues to pose a major global health challenge. By the end of 2023, an estimated 39.9 million people were living with HIV globally, with approximately 65% residing in the World Health Organization (WHO) African Region [2]. In the same year, about 1.3 million new infections and 630,000 HIV-related deaths were reported, underscoring the persistent burden of HIV, particularly in sub-Saharan Africa [2]. Pregnant women are disproportionately affected due to increased vulnerability and the risk of mother-to-child transmission [2].

HBV remains a major global health concern, affecting over 2 billion people worldwide. Despite the availability of highly effective vaccines since 1982, more than 350 million individuals continue to live as chronic carriers [3], [4]. According to the WHO 2024 report, approximately 254 million people were living with chronic HBV infection in 2022, accounting for 87% of the 1.3 million deaths attributed to viral hepatitis, largely due to cirrhosis and hepatocellular carcinoma [1] Globally, pooled prevalence among pregnant women is estimated at 4.8% for HBV and 2.9% for HIV, while in Africa, HBV prevalence is 5.89% and HIV prevalence reaches 9.6% [5]. In Ethiopia, pooled seroprevalence among pregnant women is 5.78% for HBV and 5.74% for HIV, with marked regional and sociodemographic variation [6,7]. However, local data on HBV seroprevalence and associated risk factors among pregnant women in East Africa, particularly in conflict-affected regions like Tigray, Ethiopia, remain limited [5].

Since November 2020, armed conflict in Tigray has caused extensive infrastructure damage, significant loss of life, and disruption of health services, including antenatal care (ANC) and HIV prevention programs [8]. These factors have likely increased vulnerability among pregnant women, yet data on HBV and HIV prevalence and risk factors in post-conflict Tigray remain scarce. Therefore, this study aimed to determine the seroprevalence of HBV and HIV and identify associated risk factors among pregnant women attending ANC in selected general hospitals of Mekelle and the eastern zone of Tigray, northern Ethiopia.

## Methods

### Study area, period, and design

A multicenter health facility-based cross-sectional study was conducted from December 2024 to April 2025 in three general hospitals located in Mekelle and the Eastern Zone of Tigray Region, northern Ethiopia: Mekelle General Hospital, Wukro General Hospital, and Adigrat General Hospital. Adigrat is located in the eastern zone of Tigray region at 14°16′N latitude and 39°27′E longitude, with an elevation of 2457 m above sea level [9]. Wukro is situated at 13°47′59.99″N latitude and 39°35′59.99″E longitude, and the city had an estimated population of around 50,000 in 2013 [9]. Mekelle is located approximately 784 km north of Addis Ababa and lies within the coordinates of 13°24′30″-13°36′52″N latitude and 39°25′30″-39°38′33″E longitude. The city lies in Ethiopia’s temperate highlands, with an elevation of over 2200 m above sea level [9].

### Sample size determination and sampling technique

The sample size was determined using the single population proportion method with a 95% confidence level and a 5% margin of error, based on the seroprevalence of HBV and HIV among pregnant women. An HBV seroprevalence of 10.4% was taken from a study conducted in Adigrat, northern Ethiopia [10], while an HIV seroprevalence of 7.6% was taken from a study conducted in Amhara Regional State, northern Ethiopia [11]. The HBV estimate produced the larger sample size and was therefore used as the minimum required sample. After applying a design effect of 1.5 and adding 10% for non-response, the final sample size was 238. Pregnant women attending ANC/maternal and child health clinics in the three hospitals were selected using systematic random sampling.

### Data collection procedure

In the current study, data collection was conducted using an interviewer-administered structured questionnaire along with laboratory investigations.

### Laboratory procedure for HBV and HIV screening

Each serum sample was initially screened for HBV infection using an HBsAg (HBV surface antigen) rapid test kit (HBsAg Test Kit) (Nantong Egens Biotechnology Co., Ltd., China). HBsAg-positive samples were further confirmed using the AiD™ HBsAg enzyme-linked immunosorbent assay (ELISA) (Beijing Wantai Biological Pharmacy Enterprise Co., Ltd., Beijing, China). The ELISA test for HIV was performed using a serial algorithm (three-test algorithm), which included Test 1: ONE STEP Anti-HIV (1 and 2) Test (In Tec PRODUCTS, INC., Xinyang Industrial Area, Fujian, P.R. China); Test 2: HIV 1-2. O CARD TEST (Version 2.0) RAPID HIV CARD TEST (Premier Medical Corporation Private Limited, Gujarat, India); and Test 3: Uni-Gold™ HIV Complete (Trinity Biotech Plc., IDA Business Park, Bray, Co. Wicklow, Ireland). HIV infection was further confirmed using the AiD™ HIV1+2 Ag/Ab ELISA Plus (Beijing Wantai Biological Pharmacy Enterprise Co., Ltd., Beijing, China). It is a fourth-generation ELISA that enables earlier detection of HIV by simultaneously targeting HIV-1/2 antibodies and the p24 antigen. As a result, it is capable of detecting HIV even during the window period and maintains high sensitivity (>99%) and specificity (>99%) for identifying acute infections. All tests were performed according to the manufacturers’ instructions.

### Data quality assurance

The collected data were reviewed daily for completeness and accuracy, and supervision was maintained throughout the study to ensure quality. The questionnaire was pretested and revised before data collection on 15 pregnant women at hospitals outside the study area to ensure clarity and appropriateness. Blood samples were collected, stored, and analyzed following standard procedures, with positive and negative controls included in all HBV and HIV tests.

### Statistical analysis

Data were entered and analyzed using Statistical Package for the Social Sciences version 27.0. Binary logistic regression was performed to assess the association between explanatory and outcome variables. Variables with a *P*-value ≤0.2 in the bivariate analysis were included in the multivariate logistic regression model to identify independent associations. In the presence of rare events and sparse data, exact logistic regression analysis was fitted using STATA to generate robust and valid estimates for HIV seroprevalence, with model adequacy assessed based on overall model convergence and exact likelihood ratio statistics. Adjusted odds ratios (AORs) with 95% confidence intervals (CIs) were calculated, and statistical significance was set at *P* < 0.05.

### Ethical considerations

The study was conducted after obtaining ethical clearance from the Ethical Review Committee of the College of Medicine and Health Sciences, Mekelle University. Formal permission was also obtained from the Tigray Regional Health Bureau and the administrative offices of each participating hospital. Verbal informed consent was obtained from all study participants. Participants who tested positive for HBsAg or HIV were referred to physicians for appropriate care and antenatal services. All information collected during the study was kept confidential, and clinical specimens were used solely for the objectives of this study

## Results

### Sociodemographic characteristics

A total of 238 pregnant women participated in the current study. The average age of the pregnant women was 27.3 ± 5.5 years**,** with the majority **(**62.2%**)** being older than 25 years. A significant proportion (95.0%) of the women were married, with only small fractions being single (3.8%) and divorced (1.3%). More than three-quarters of the women (76.9%) resided in urban areas, and nearly half (49.6%) were housewives. Regarding education, secondary level was the most common (42.9%), followed by tertiary (26.9%) and primary (23.1%) levels of education. Table 1 further illustrates the socioeconomic and family contexts of the participants**.**

**Table 1:** Sociodemographic characteristics of pregnant women attending ANC in three selected hospitals, northern Ethiopia (N = 238).

Variable	Category	Number	Percent (%)
ANC follow-up site	Adigrat General Hospital	79	33.2
	Wukro General Hospital	75	31.5
	Mekelle General Hospital	84	35.3
Age (years)	≤25 years	90	37.8
	>25 years	148	62.2
Marital status	Single	9	3.8
	Married	226	95.0
	Divorced	3	1.3
Residence	Rural	55	23.1
	Urban	183	76.9
Educational level	Illiterate	17	7.1
	Primary	55	23.1
	Secondary	102	42.9
	Tertiary	64	26.9
Family size	1-3	119	50.0
	4-6	108	45.4
	>6	11	4.6
Current occupation	Housewife	118	49.6
	Farmer	27	11.3
	Merchant	45	18.9
	Private employee	5	2.1
	Government employee	43	18.1
Working in a health facility (N = 48)	No	44	91.7
	Yes	4	8.3
Monthly income (in ETB)	≤3000	154	64.7
	3000-6000	55	23.1
	6000-9000	13	5.5
	>9000	16	6.7
History of tattoo	No	210	88.2
	Yes	28	11.8

Abbreviations: ANC, antenatal care, ETB, Ethiopian Birr.

### Seroprevalence of HBV and HIV

**Figure 1:**
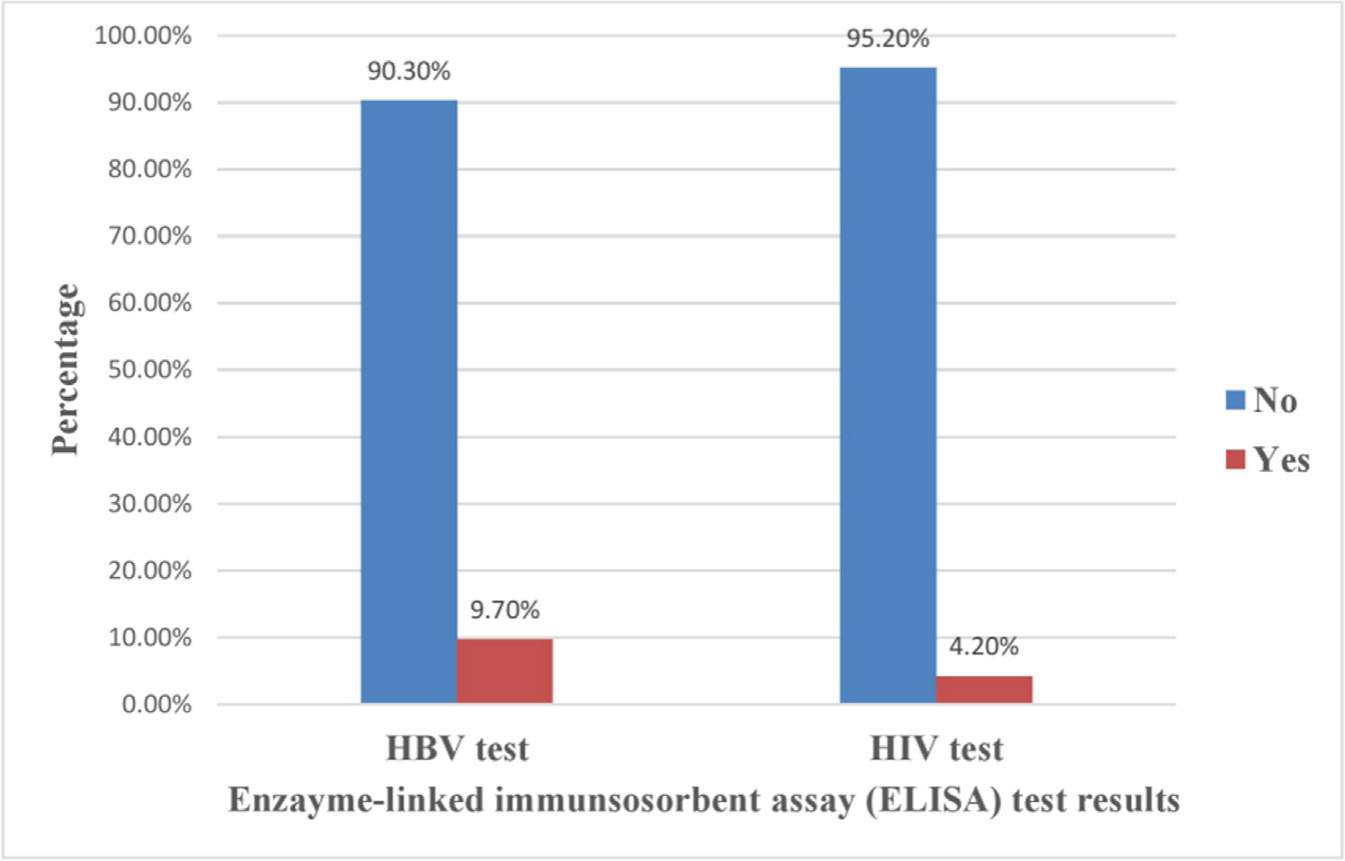
Bar charts illustrating the prevalence of HBV (left) and HIV (right) among pregnant women attending antenatal care (ANC) at three selected hospitals in Northern Ethiopia (N = 238).

Out of 238 pregnant women screened using serological and ELISA techniques, 23 women (9.7%) tested seropositive for HBV (Figure 1). Additionally, 10 women (4.2%) tested positive for HIV. However, no cases of HBV/HIV co-infection were identified, indicating a zero HBV/HIV co-infection rate. The seroprevalence of HBV in Mekelle, Adigrat, and Wukro was 11.9%, 8.9%, and 8%, while the seroprevalence of HIV was 6%, 3.7%, and 2.7%, respectively.

### Behavioral and knowledge related to HBV and HIV transmission

Notably, a vast majority of the women reported no history of smoking (100%) or alcohol consumption (98.7%). Most also indicated no history of multiple sexual partners (94.5%), sharing sharp instruments (92.9%), blood transfusion (100%) and sexual violence (98.7%), In addition, a large proportion had no prior liver disease (97.5%). In terms of knowledge and health-related factors, a significant proportion lacked awareness about HBV transmission modes (85.3%), whereas the majority demonstrated knowledge of HIV transmission (94.1%). Furthermore, 24.4% of the women reported a history of abortion and nose piercing (22.7%) (Table 2).

**Table 2:** Behavioral and knowledge related to HBV and HIV of pregnant women attending antenatal care in three selected hospitals, northern Ethiopia (N = 238).

Variable	Category	Number	Percent (%)
History of smoking	No	238	100.0
	Yes	0	0.0
History of alcohol drinking	No	235	98.7
	Yes	3	1.3
History of multiple sexual partners	No	225	94.5
	Yes	13	5.5
Knowledge about HBV transmission	No	203	85.3
	Yes	35	14.7
Knowledge about HIV transmission	No	14	5.9
	Yes	224	94.1
History of sharing common sharp materials	No	221	92.9
	Yes	17	7.1
History of blood transfusion	No	238	100.0
	Yes	0	0.0
History of surgical procedures	No	224	94.1
	Yes	14	5.9
History of sexual violence	No	235	98.7
	Yes	3	1.3
History of unprotected sexual practice	No	236	99.2
	Yes	2	0.8
HBV vaccination	No	232	97.5
	Yes	6	2.5
History of liver disease	No	232	97.5
	Yes	6	2.5
History of abortion	No	180	75.6
	Yes	58	24.4
History of nose piercing	No	184	77.3
	Yes	54	22.7
History of STIs	No	224	94.1
	Yes	14	5.9

Abbreviations: HBV, hepatitis B virus; HIV, human immunodeficiency virus; STI, sexually transmitted infection.

### Associated risk factors for HBV and HIV infection

The bivariate logistic regression analysis identified six candidate variables for HBV seroprevalence**:** age, educational level, HBV vaccination status**,** history of liver disease, history of abortion, nose piercing practice, and history of sexually transmitted infections (STIs)**.** However, HBV vaccination status was excluded from the final model due to a violation of the chi-square assumption, and the final multiple logistic regression model was run with the remaining six variables. Multivariate logistic regression analysis was conducted to assess independent risk factors for HBV infection. Older age (>25 years) was linked to higher HBV positivity (AOR = 3.10, 95% CI: 1.01-9.46, *P* = 0.047), likely due to prolonged exposure. The history of liver disease showed the strongest correlation (AOR = 23.67, 95% CI: 3.55-158.01, *P* = 0.001), reinforcing its role in HBV susceptibility. Additionally, nose piercing (AOR = 3.80, 95% CI: 1.33-10.88, *P* = 0.013) and history of STIs (AOR = 6.03, 95% CI: 1.56-23.35, *P* = 0.009) significantly increased the odds of HBV infection. Interestingly, the history of abortion was inversely associated with HBV positivity (AOR = 0.24, 95% CI: 0.06-0.98, *P* = 0.047) (Table 3).

**Table 3:** Associated possible risk factors related to HBV seroprevalence among pregnant women attending antenatal care in three selected hospitals, northern Ethiopia (N = 238).

Variable	Category	HBsAg (%)	COR	*P*-value	AOR (95% CI)	*P*-value
Age (years)	≤25 years	5 (5.6)	1.00		1.00	
	>25 years	18 (12.2)	2.35	0.103	3.10 (1.01-9.46)	**0.047**^a^
Educational level	Illiterate	2 (11.8)	2.00	0.448	2.20 (0.32-15.19)	0.424
	Primary	8 (14.6)	2.55	0.145	3.21 (0.81-12.73)	0.097
	Secondary	9 (9.9)	1.45	0.550	1.57 (0.42-5.93)	0.503
	Tertiary	4 (6.2)	1.00		1.00	
History of liver disease	No	20 (8.6)	1.00		1.00	
	Yes	3 (50.0)	10.6	0.005	23.67 (3.55-158.01)	**0.001^b^**
History of abortion	No	20 (11.1)	1.00		1.00	
	Yes	3 (5.2)	0.44	0.194	0.24 (0.06-0.98)	**0.047**^a^
History of nose piercing	No	15 (8.2)	1.00		1.00	
	Yes	8 (14.8)	1.96	0.151	3.80 (1.33-10.88)	**0.013**^a^
History of STIs	No	19 (8.5)	1.00		1.00	
	Yes	4 (28.6)	4.32	0.022	6.03 (1.56-23.35)	**0.009**^b^

Note: 1.00: reference category.

Abbreviations: AOR, adjusted odds ratio; CI, confidence interval; COR, crude odds ratio; HBsAg, hepatitis B virus surface antigen; HBV, hepatitis B virus; STI, sexually transmitted infection.

a *P* < 0.05. Considered as statisticaly significant

b *P* < 0.01 indicates srongly ststisticaly significant

### Factors associated with HIV

Exact logistic regression identified a significant association between HIV knowledge and reduced HIV seroprevalence, with informed women showing 90% lower odds of infection (exact odds ratio [OR] = 0.10, 95% CI: 0.02-0.72, *P* = 0.0219). Although higher odds were observed among women in the third trimester and those who reported sharing sharp utensils, multiparous women had lower odds compared to nulliparous/primiparous women; however, none of these associations were statistically significant. The findings underscore the protective role of HIV-related awareness among pregnant women (Table 4).

**Table 4:** Exact logistic regression analysis to identify factors associated with HIV seroprevalence among pregnant women attending antenatal care in three selected hospitals, northern Ethiopia (N = 238).

Variable	Category	Anti-HIV (%)	Exact OR (95% CI)	Exact *P*-value
Knowledge about HIV transmission	No	3 (21.4)	1.00 (reference category)	
	Yes	7 (3.1)	0.10 (0.02-0.72)	**0.0219**^a^
Stage of pregnancy	First/second	6 (3.2)	1.00 (reference category)	
	Third	4 (8.0)	3.63 (0.67-18.58)	0.1444
Parity	Nulliparous/primiparous	7 (5.7)	1.00 (reference category)	
	Multiparous	3 (2.6)	0.38 (0.06-1.78)	0.2854
Sharing common sharp utensils	No	8 (3.6)	1.00 (reference category)	
	Yes	2 (11.8)	5.51 (0.43-47.84)	0.2041

Abbreviations: Anti-HIV, antibodies against human immunodeficiency virus; CI, confidence interval; HIV, human immunodeficiency virus; OR, odds ratio.

a *P* < 0.05 considered significant.

## Discussion

In our study, the seroprevalence of HBsAg and HIV among pregnant women attending ANC in three general hospitals of Tigray was 9.7% and 4.2%, respectively. Mekelle General Hospital reported the highest prevalence, followed by Adigrat and Wukro. According to the Centers for Disease Control and Prevention criteria, the HBV prevalence classifies the area as highly endemic (>8%) [12]. Our HBV prevalence was comparable to findings from Adigrat, Gedeo, Jigjiga, Sudan, and Gambia (8.5-9.2%) [[13], [14], [15], [16], [17]], higher than reports from Borena, Amhara, Nekemte, and Bishoftu (4.5-6.5%) [11,[18], [19], [20]], and lower than studies conducted in some African countries, such as Nigeria [21] and Ghana [22], which reported prevalences of 17.2% and 16.7%, respectively. These differences might be due to demographic variation across study areas, differences in study periods, variation in exposure to behavioral practices, sample size, and laboratory technique used for sample analysis.

In this study, pregnant women with a history of nose piercing were four times more likely to be infected with HBV compared to those without such a history. Similarly, a previous history of STIs was significantly associated with HBV infection; women with a prior STI were six times more likely to be infected than those without a history of STIs. This finding is consistent with a study conducted in the Borena Zone, southern Ethiopia [18], in 2022, which reported that a history of STIs was associated with HBV infection (AOR = 5.99, 95% CI: 1.81-19.85) among pregnant women attending ANC at public hospitals.

Additionally, pregnant women older than 25 years were three times more likely to be infected with HBV than younger women. This association between older age and HBV infection contrasts with a study conducted in the Gedeo Zone, southern Ethiopia [14], which reported that age younger than 20 years was significantly associated with HBV infection (AOR = 5.1, 95% CI: 1.5-18.0). This discrepancy may be due to differences in sexual and behavioral practices among pregnant women in the respective study areas.

Other factors associated with HBV infection in our study included a history of abortion and liver disease. These findings differ from a study conducted in Jigjiga [15], which identified previous surgical procedures, family history of HBV, sharing sharp materials, and multiple sexual partners as significant risk factors. The observed discrepancies may be attributed to variations in study populations, cultural practices, and exposure to risk behaviors.

The seroprevalence of HIV among pregnant women attending ANC in our study was 4.2%, slightly lower than the pooled prevalence reported among pregnant women in Ethiopia, which is 5.74% (95% CI: 3.96-7.53%) [7]. It was also similar to regional estimates from Addis Ababa (4.80%, 95% CI: 3.12-6.49) and Oromia (4.48%, 95% CI: 2.56-6.41) [7], and to a study conducted in Wolaita Sodo (3.8%) [23]. In contrast, our prevalence was higher than reports from Simada Hospital, South Gondar Zone, and a rural hospital in southern Ethiopia, which reported 2% and 1.8%, respectively [24,25], but lower than findings from Jimma University Specialized Hospital (7.1%), the Amhara region (7.6%), Bahir Dar (6.6%), and Gondar Health Center (11.9%) [11,[26], [27], [28]]. These variations may reflect differences in geographical location, HIV awareness and education, cultural and behavioral practices, access to ANC services, and differences in study design and sample size.

Additionally, knowledge about HIV transmission was significantly associated with HIV seroprevalence among the study participants (exact OR = 0.10, 95% CI: 0.02-0.72, *P* = 0.0219), suggesting that greater awareness may reduce infection risk. This finding contrasts with the study conducted at Simada Hospital [24], which reported that sharing sharp materials, history of dental procedures, abortion, and blood transfusion were significantly associated with HIV infection in bivariate analysis. The differences could be due to variations in participant demographics, HIV knowledge and awareness, cultural and behavioral practices, and access to ANC and HIV prevention services.

Piercing and the sharing of sharp instruments with unsterile materials remain common practices in many parts of Ethiopia [27], which can increase the risk of HIV infection through exposure to contaminated blood. In our study, the association between sharing sharp utensils and HIV seropositivity was not statistically significant. In contrast, a study conducted in Bahir Dar [27] reported a significant association between HIV infection and piercing with sharp instruments (AOR = 3.0, 95% CI: 1.17-7.80) as well as a history of abortion (AOR = 6.6, 95% CI: 2.50-17.71). The observed differences may be explained by variations in access to safe procedures, sample size, and adjustment for potential confounding factors.

Women in their third trimester had higher odds of HIV infection (exact OR = 3.63, 95% CI: 0.67-18.58, *P* = 0.1444), and those who shared sharp utensils also showed higher odds (exact OR = 5.5, 95% CI: 0.43-47.84; *P* = 0.2041). Women who had previously given birth (multiparous) appeared to have lower odds (exact OR = 0.38, 95% CI: 0.06-1.78; *P* = 0.2854). However, these results are uncertain because the number of HIV-positive women was small and the CIs were wide, so the findings may not fully reflect the true risk. More research with larger groups of pregnant women is needed to confirm these results.

Although the prevalence of HBV and HIV was relatively high among the study participants, no HIV/HBV co-infection was identified in this study. This finding aligns with reports from eastern Ethiopia, where a co-infection rate of 0.0% was observed [29]. The absence of co-infection in our study could be due to differences in the timing of infection and host immune response, which may limit the overlap of HIV and HBV in pregnant women. However, this contrasts with higher co-infection rates reported in other regions, such as Bahir Dar City (19%) [27], and was slightly lower than that reported in a study from southern Ethiopia (0.6%) [25]. These discrepancies may also reflect variations in study design, sample size, and local cultural or behavioral practices affecting transmission.

In our study, limited laboratory capacity prevented the assessment of additional HBV markers, such as HBeAg, HBcAg, molecular confirmation using HBV deoxyribonucleic acid, and HIV ribonucleic acid viral load among women living with HIV, representing key limitations.

## Conclusion

The overall seroprevalence of HBV among pregnant women attending ANC was high according to the WHO classification. The seroprevalence of HBV and HIV was highest in Mekelle General Hospital and lowest in Wukro General Hospital. Risk factors, including older age, history of liver disease, previous history of abortion, history of nose piercing, and history of STI, were significantly associated with seroprevalence of HBV, while knowledge about transmission of HIV was significantly associated with seroprevalence of HIV.

Based on the study findings, efforts should focus on increasing awareness of HBV and HIV transmission among pregnant women through targeted training, public health campaigns, and social media. Continuous follow-up and counseling are recommended to address high-risk behaviors and ensure proper aseptic practices during clinical procedures. Pregnant women at high risk should receive regular medical checkups and professional guidance and be encouraged to deliver in health facilities. Additionally, those who test positive for HBV or HIV should adhere strictly to prescribed treatments to prevent mother-to-child transmission and improve maternal and neonatal outcomes.

## Declaration of competing interest

The authors have no competing interests to declare.
